# A New and All-Solid-State Potentiometric Aluminium Ion Sensor for Water Analysis

**DOI:** 10.3390/s20236898

**Published:** 2020-12-03

**Authors:** Kook Shih Ying, Lee Yook Heng, Nurul Izzaty Hassan, Siti Aishah Hasbullah

**Affiliations:** Department of Chemical Sciences, Faculty of Science and Technology, Universiti Kebangsaan Malaysia, 43600 Bangi, Selangor, Malaysia; kookshihying@siswa.ukm.edu.my (K.S.Y.); yhl1000@ukm.edu.my (L.Y.H.); drizz@ukm.edu.my (N.I.H.)

**Keywords:** all-solid-state detection system, potassium ion-selective electrode, aluminium sensor, poly(*n*-butyl acrylate) membrane

## Abstract

An all-solid-state potentiometric electrode system for aluminium ion determination was developed with a new aluminium ion sensor as the working electrode based on a new ionophore for aluminium ion, 1,1′-[(methylazanediyl)bis(ethane-2,1-diyl)]bis[3-(naphthalen-1-yl)thiourea] (ACH). The reference electrode was a potassium ion sensor, which acts as a pseudo-reference. Both electrodes were made from Ag/AgCl screen-print electrodes fabricated from a non-plasticized and photocurable poly(*n*-butyl acrylate) membrane that contained various other membrane components. The pseudo-reference potential based on the potassium ion sensor was fixed in 0.050 M KNO_3_, and such concentration of K^+^ ion did not interfere with the measurement of the Al^3+^ ion using the aluminium sensor. With such a pseudo-reference and in the presence of 0.050 M KNO_3_ as a background medium, the aluminium sensor measured changes of aluminium ion concentrations linearly from 10^−6^ to 10^−2^ M Al^3+^ ion with a Nernstian response of 17.70 ± 0.13 mV/decade. A low detection limit of 2.45 × 10^−7^ M was achieved with this all-solid-state potentiometric system. The aluminium sensor was insensitive to pH effects from 2.0 to 8.0 with a response time of less than 50 s. Under optimum conditions, a lifetime of 49 days was achieved with good sensor selectivity, reversibility, repeatability, and reproducibility. The all-solid-state electrode system was applied to analyze the Al^3+^ ion content of water samples from a water treatment plant. Compared with the conventional potentiometric detection system for aluminium ions, the new all-solid-state aluminium ion sensor incorporating a pseudo-reference from the potassium sensor demonstrated similar analytical performance. It thus provided a convenient means of aluminium content analysis in water treatment plants.

## 1. Introduction

Aluminum in its naturally occurring form is stable and does not interact with biological systems. However, its soluble form released via rocks and soil into the acidic environment can be absorbed by plants and animals [1]. High aluminium levels were reported to adversely influence fish, algae, and crops [2,3]. Besides, aluminium in excessive levels can cause many diseases such as Parkinson’s disease, Alzheimer’s disease, bone softening, lung function decline, and chronic kidney failure [4]. The daily human aluminium consumption ranges between 2.5 and 13.0 mg, according to the World Health Organization (WHO) [5], whereas for drinking water, the allowable level of aluminium is 0.20 ppm [6,7]. Due to its occurrence in the environment and its toxic nature, it is even more critical to identify and determine its trace quantities, therefore, a simple but efficient method for aluminium monitoring is highly required.

A potentiometric method is an attractive analytical method due to its reasonable selectivity, simple construction, ability to determine ions in a wide linear range, and beneficial in the environmental field [8,9]. The miniaturization trend has increased over the past decades, thus placing it at the forefront of in situ electrochemical analysis methods [10]. This method also offers advantages, such as analyzing small sample sizes, prompt analysis, ideal for automation procedures, and excellent repeatability [8]. Therefore, as an efficient analysis tool, it is suitable for the determination of aluminium ions.

Miniaturization of an ion-selective electrode can be achieved easily via thick-film, thin-film, and microcavity techniques [11]. However, the fabrication of a reliable solid-state reference electrode from a conventional type one remains a crucial challenge for the construction of sufficiently miniaturized potentiometric systems [12,13]. The conventional reference electrode contains a concentrated aqueous equitransferrent salt solution referred to as a salt bridge. It acts to reduce the bridge electrolyte’s liquid interface potential with the samples [14], thus providing a stable and sample-independent potential. To fabricate a reference electrode using a solid-state process, a suitable replacement of that function is the primary consideration, and remains a challenge in this field.

Generally, all-solid-state reference electrodes rely on a solid contact layer (internal electrolyte replacement) sandwiched between an underlying electron-conducting substrate and a hydrophobic reference membrane. The solid contact layer is the internal electrolyte replacement and formed from a gel-like electrolyte or molten salts such as potassium chloride [15], single-walled carbon nanotubes [10], colloid-imprinted mesoporous materials [14], and conducting polymers like polypyrrole [16] and poly(3,4-ethylene dioxythiophene) [17]. The samples do not influence the interfacial potential at the membrane/solid contact interface and involve the electrical double layer stabilizing redox reactions of a conductive polymer or solid contacts interface [12]. 

Membranes of reported reference electrodes generally help in shielding the transducer from interferences. They are usually doped with salts such as ionic liquids, cation, and anion lipophilic and solid salts to create a constant potential [13,14,16,18]. The interfacial potential at the membrane-sample interface depends on the local distribution of the doping ions across these two immiscible phases. It is not influenced by the sample [12]. Therefore, to produce a stable and reproducible sample-independent potential, the selection of the solid contact and the formulation of a reference membrane are the two essential parameters in the design of a solid-state reference electrode. 

Many designs and trials were described for the fabrication of solid-state type reference electrodes; for example, Simonis et al. [15] described a solid-state reference electrode where the Ag/AgCl electron-conducting layers were coated with agar containing 0.5 M KCl salts and then covered with poly(vinyl chloride)/cellulose nitrate (PVC/SN). The agar with 0.5 M KCl was utilized as the replacement of an internal filling solution. However, a high concentration of 0.5 M KCl applied directly onto the surface of Ag/AgCl caused AgCl dissolution. The dissolution usually occurs in a thin-film electrode with such design as the low quantity of AgCl on the electrode can be dissolved entirely into the salt electrolyte. Thus, the reference electrode becomes unstable and has a short shelf life [19,20]. This reference electrode was combined with a thin-film pH sensor to form a single chip sensor. However, a sub-Nernstian response of 41 mV/decade was obtained in the pH range from 5 to 7. 

This initial idea of solid-state reference electrode was then refined by Maminska et al. [21], where a planar Ag/AgCl microelectrodes with PVC membranes consisting of the ionic liquid 1-dodecyl-3-methyl-imidazolium chloride (IL) was used. The IL-additive membrane provides an internal solid electrolyte to keep the chloride concentration within the layer constant, thus creating a constant potential of the solution’s electrode. When used with a potassium ion sensor, an excellent Nernstian response was obtained at 54.5 mV/decade. It showed a comparable result with a conventional reference electrode (55.0 mV/decade). However, Rius-Ruiz et al. [13] reported that such electrode design might experience undesirable signal disturbances caused by Ag^+^ ion in the system and the lipophilic species. 

Conducting polymer (CP), an ion-to-electron transducer [22], was reported as the replacement of the internal electrolyte due to solid transducers are well-known to provide necessary stability to potentiometric electrodes [13]. A solid-state reference electrode based on the CP, poly(3,4-ethylendioethylene), and polypyrrole doped with anion (4-styrene sulfonic) anion was reported by Kisiel et al. [16,17]. The CP layer of both electrodes was then coated with a PVC membrane with a dispersion of Ag, AgCl, and KCl salts to yield insensitive potentiometric responses. However, the combination of CP and membrane faced problems of potential change and low repeatability. It appeared when the CP layer was not sufficiently attached to the membrane. Besides that, an inner water layer favors the formation between the CP and the membrane interface [23]. The second type of reference electrode was used together with lead ion sensor (29.9 mV/decade in the linear range of 10^−5^ to 10^−2^ M) and calcium ion sensor (27.3 mV/decade in the linear range of 10^−4^ to 10^−1^ M). However, both the Nernstian responses were displayed at a narrow linear range. A super-Nernstian response was even obtained at the concentration range of 10^−6^ to 10^−4^ M Ca^2+^.

For the reference membrane, most of the reported solid-state reference electrodes have utilized a plasticized polymer matrix such as a PVC membrane [14,16,17,18,21], where plasticizer is required to obtain optimum response [24,25]. The addition of plasticizers could nevertheless reduce the polymer’s mechanical strength and adhesion on the electrode surface. It is easy to be peeled off from the transducer and induces a drastic change in sensors response, thus shortening the transducer [26,27]. Furthermore, water intake occurred in a highly plasticized PVC membrane. Lindfors et al. [28] recorded that a high amount of plasticizer within the polymer membrane facilitates water intake. This observation was due to the membrane with a high concentration of plasticizer with low glass transition temperature value (Tg), contributing to lower resistance, hence enhancing the transfer of water into the membrane [27]. 

The polyacrylic polymer is reported as an alternative kind of polymeric membrane today in solid-state electrodes. It has better analytical performance characteristics over the plasticized membrane due to its low ion and water diffusion coefficients. Among the polyacrylate membranes, poly(*n*-butyl acrylate), the pBA membrane was selected because of its capability to produce membranes with lower water percolation and good characteristics [10]. An Ag/AgCl solid-state screen-printed reference electrode based on photocurable acrylic membrane with immobilized trioctylmethy ammonium chloride (TOMA-Cl) and sodium tetrakis [3,5-bis(trifluoromethyl)phenyl]-borate (NaTFPB) was reported by Alva et al. [29]. It gave performances similar to those of conventional reference electrodes when used with potasium and ammonium ion sensors. However, it still faced significant interference from perchlorate (ClO_4_^-^) ions. Besides that, using two lipophilic salts to control the charge balance within the membrane faced some limitations. One of the lipophilic salts tended to leach out into the sample from the acrylic membrane. Therefore, this caused a charge imbalance and instability of the reference electrode [20]. 

All the efforts mentioned above are interesting from the scientific point of view, but further developments are still required to improve their practical applicability. The particular miniaturized reference electrodes’ design experienced some shortcomings, especially in terms of stability, interference, and lifetime. Some solid-state reference electrodes also showed a sub-Nernstian response and short linear range when performed together with ion sensors. 

In this work, instead of fabricating a solid-state reference electrode using various membrane designs as reported in the past, we attempted to use a solid-state ion-selective electrode as a pseudo-reference. The constant potential requirement is achieved by exposing the pseudo-reference in a fixed concentration of the pseudo-reference’s primary ions. Coupled with a solid-state aluminium ion sensor, we could develop an all-solid-state potentiometric system to measure aluminium ions. Among the various types of ion sensors, the potassium ion sensor was selected as a pseudo-reference electrode. This is based on several reasons, such as the potassium ionophore, valinomycin, which was well established [27,30].

Furthermore, potassium ions do not significantly interfere with the aluminium ion sensor used as a working electrode [31]. Another consideration is the non-toxic nature of potassium ions [32,33]. Thus, a solid-state potassium sensor’s suitability as a pseudo-reference for aluminium ion sensor determination was first assessed. 

## 2. Materials and Methods

### 2.1. Chemicals

For membrane preparation, *n*-butyl acrylate (nBA) from Merck, 2-hydroxyethyl methacrylate (HEMA), 1,6-hexanediol diacrylate (HDDA), 2-dimethoxy-2-phenyl acetophenone (DMPP), sodium tetrakis [3,5-bis(trifluoromethyl) phenyl] borate (NaTFPB), potassium tetrakis-(4-chlorophenyl)borate (KTClPB) and potassium ionophore I from Sigma Aldrich (St. Louis, MO, USA) were used. The solvents and the metal salts included potassium chloride, copper (II) chloride dihydrate, calcium chloride and sodium chloride from Sigma Aldrich, cadmium chloride and aluminium chloride from Acros, mercury chloride from Fisher, zinc chloride and nickel chloride hexahydrate from Merck, iron(III) chloride from R&M Chemicals, tetrahydrofuran from SYSTERM and magnesium nitrate from Bendosan. Lithium acetate dihydrate from Acros, tris(hydroxymethyl)aminomethane hydrochloride, Tris-HCl, and silver chloride from Sigma Aldrich and bacto agar from Difco used for conventional reference electrode preparation. The chemicals used were the aluminium ionophore 1,1′-[(methylazanediyl)bis(ethane-2,1-diyl)]bis[3-(naphthalen-1-yl)thiourea], ACH synthesized and purified according to Kook et al. [34] and Abosadiya et al. [35]. All analytical grade chemicals were used as obtained. Deionized water (specific resistance, 18.0MΩ cm) was utilized throughout this work. 

### 2.2. Instrumentations

The conventional reference electrode for the potentiometric measurement was a standard double junction Ag/AgCl. For this electrode, the internal reference solution was 0.1 M Tris-HCl buffer at pH 7.00 saturated with AgCl, and the gel bridge electrolyte was 0.1 M lithium acetate. Screen printed Ag/AgCl electrode (Scrint Print Sdn. Bhd., Kedah, Malaysia) was used to manufacture potassium and aluminium ion-selective electrodes. An Orion Versa Star Advanced Electrochemical Meter (Thermo Fisher Scientific, Waltham, MA, USA) was used to perform electrochemical measurements. Four-light tubes were installed into the ultraviolet (UV) exposure unit (R.S. Ltd., Cambridge, UK) to transmit a UV wavelength of 350 nm for photopolymerization.

### 2.3. Preparation and Deposition of Poly(2-hydroxylethyl methacrylate) (pHEMA) and Ion-Selective Poly(n-butyl acrylate) (pBA) Membrane

#### 2.3.1. Preparation of Aluminium Ion Sensor

**Figure 1:**
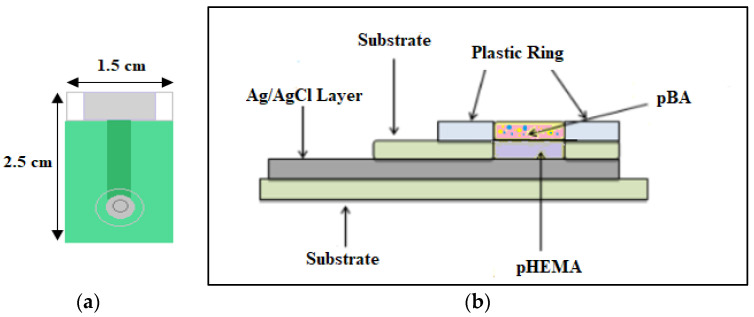
(**a**) A screen-printed Ag/AgCl electrode used for the design of both pseudo-reference (potassium ion sensor) and aluminium ion sensor (**b**) A cross-section and side view of the screen-printed electrode.

The aluminium ion sensor was fabricated according to Kook [31]. The self-plasticizing polymers, pHEMA, and pBA were both through a photopolymerization process. For aluminium ion sensor fabrication, 0.1 µL of HEMA monomer, which consisted of 1.6 wt% of photoinitiator DMPP, was deposited on the tip of the screen-printed Ag/AgCl electrode. Subsequently, the electrode was fixed in the UV-exposure unit. Nitrogen gas was purged for 2 min and then irradiated for another 3 min. The polymer film, pHEMA formed, was hydrated with 0.01 M of aluminium chloride solution for 15 min to form the “inner solution”. A subsequent monomer stock solution of nBA was prepared with a mixture of 950 μL of nBA and 1 μL of HDDA to form the ion-selective poly(nBA) membrane. The Eppendorf tube consisted of 1 mg DMPP, and 100 μL of that stock solution was prepared. 2 μL of the mixture was dropped onto the pHEMA-SPE and underwent photopolymerization under UV exposure for 6 min. For the last step, 10 mol% lipophilic salt, NaTFPB, and 3.0 mg of ionophore ACH were dissolved in 100 µL of THF. 2 µL of the stock solution (two portions with 1 µL each) was dropped onto pBA-pHEMA-SPE, and the electrode was left overnight in room temperature to evaporate the THF. Figure 1 illustrates the design of pBA-pHEMA-screen-printed Ag/AgCl electrodes.

#### 2.3.2. Preparation of Potassium Ion Sensor (Pseudo-Reference Electrode)

The ion sensor developed with minor modification following the method previously described [32,36,37]. The pHEMA membrane was prepared following the procedure in Section 2.3.1. The hydration solution used was changed to 0.1 M potassium nitrate for 30 min. Another stock solution of monomer nBA was prepared for the formation of ion-selective poly(nBA) membrane by mixing 950 µL of nBA and 1 µL of cross-linker HDDA. 100 µL of that stock solution added to an Eppendorf tube consisted of 1.5 mg of potassium ionophore I, 60 mol% of anion lipophilic salt, and KTClPB and 1 mg of DMPP. 2 μL of the mixture was dropped onto the pHEMA-SPE and underwent photopolymerization under UV exposure for 8 min. The mixture was kept at 4 °C when not in use. 

#### 2.3.3. Evaluation of the Potassium Ion Sensor 

The sensitivity of the potassium ion sensor was measured in a series of standard potassium nitrate solutions in a range from 10^−8^ to 10^−1^ M. The measurements consisted of a complete electrochemical cell with a double junction conventional Ag/AgCl reference electrode and ion sensor connected to the Orion meter. The stable potential difference or electromotive force (emf) in the mV unit was obtained and recorded. The emf graph versus the logarithm of potassium ion concentration was plotted, and the slope was determined. Separate solution method (SSM) was used to determine the selectivity of the potassium ion sensor. 0.1 M of interference ions such as KCl, NaCl, CuCl_2_, CdCl_2_, HgCl_2_, NiCl_2_, CaCl_2_, ZnCl_2_, Pb(NO_3_)_2_, Mg(NO_3_)_2_, and FeCl_3_ were prepared for the study [38]. For the reversibility study, the same electrode was measured from 10^−8^ to 10^−1^ M K^+^, followed by 10^−1^ to 10^−8^ M K^+^ and finally from 10^−8^ to 10^−1^ M K^+^. The repeatability analysis was conducted by measuring the same electrode thrice from 10^−8^ to 10^−1^ M K^+^. Whereas for the reproducibility study, three different batches of electrodes were measured. The stability of the electrode was evaluated through the drift of the potential value per time. The electrode was immersed together with a double junction conventional Ag/AgCl reference electrode in 0.050 M potassium nitrate solution for 24 h. The measurement of the potential value was executed hourly.

### 2.4. Assessment of the All-Solid-State Aluminium Ion Sensor System 

#### 2.4.1. Potentiometric Measurements

The all-solid-state system’s response having a potassium ion sensor as a pseudo-reference electrode for aluminium ion sensor was tested in a series of standard solutions of aluminium chloride in the range of 10^−8^ to 10^−1^ M with the addition of a constant concentration of KNO_3_. A complete electrochemical cell consisting of a pseudo-reference electrode and aluminium ion sensor connected to an Orion meter was assembled during the measurements. The emf value was recorded when a stable reading was reached. The graph of emf versus the logarithm of aluminium ion concentration was plotted to determine the graph’s slope.

Selectivity studies were performed to determine potassium or aluminium ion sensors’ ability to select the primary ions over the interference ions. In the potassium ion sensor’s case as a pseudo- reference, a good selectivity could prevent potential shifting. The selectivity study was carried out using the separate solution method (SSM) to determine the sensor’s logarithm selectivity coefficient via the Nicolsky-Eisenmann equation below:

(1) $${\mathrm{logK}}_{A,\;B}^{\mathrm{pot}}=\frac{\left(E_{B\;}–E_{A\;}\right)Z_{A\;}F}{2.303\mathrm{RT}}\;+\left(1-\frac{Z_{A}}{Z_{B}}\;\right){\mathrm{loga}}_{a}$$

This method was utilized when the sensor revealed the response of Nernstian within a linear range. Based on the equation, if the ${\mathrm{logK}}_{A,\;B}^{\mathrm{pot}}$ give a smaller value (below 1); the sensor is expected to be more responsive to the primary ions (A) than the interference ion (B). Therefore, the smaller the value of the ${\mathrm{logK}}_{A,\;B}^{\mathrm{pot}}$, the more selective the sensor towards the primary ion A [38]. 

#### 2.4.2. Optimization of the Performance of the All-Solid-State Aluminium Ion Detection System

A series of KNO_3_ concentrations (0.500, 0.250, 0.100, 0.075, 0.050, 0.025, 0.010, 0.005 and 0.001 M) was added in the standard solutions of aluminium chloride in the range from 10^−8^ to 10^−1^ M to determine the optimum concentration for potassium ion sensor to have functioned as a pseudo-reference electrode with a constant potential. The effect of each concentration of KNO_3_ on the response of the sensor was studied. 

A series of 10^−3^ M AlCl_3_ and 0.050 M KNO_3_ solutions with a pH range from 1.0 to 9.0 were prepared, and the effect of the pH towards the sensor response measured. The pH of each solution was adjusted with 1 M NaOH and 1 M HNO_3_. For reversibility, repeatability, and reproducibility studies, 0.050 M KNO_3_ was added in each concentration of standard solutions of AlCl_3_ from 10^−8^ to 10^−1^ M. For stability study, 0.010 M of AlCl_3_ and 0.050 M KNO_3_ were prepared in deionized water. The measurement of reversibility, repeatability, reproducibility, and stability studies was conducted as procedure 2.3.3. The pseudo-reference electrode response time with an aluminium ion sensor was investigated in standard solutions of 10^−8^ to 10^−1^ M Al^3+^ with added 0.050 M KNO_3_ in each standard solution. The response time was described when the two electrodes were immersed in the solution until a stable reading was achieved for several minutes. The detection limit was calculated from the intersection of two extrapolated segments of the calibration curve [39]. For the selectivity study, 0.010 M of interference ions KCl, NaCl, CaCl_2_, Mg(NO_3_)_2_, and FeCl_3_ with 0.050 M KNO_3_ in each solution were used. These interference ions have been selected because of their abundance in the water samples. The shelf life was examined in standard solutions of aluminium chloride in 10^−8^ to 10^−1^ M with 0.050 M KNO_3_ over days. Thirty batches of the pseudo-reference electrode and aluminium ion sensor were prepared and stored in dry, closed, and room temperature while not in use. Three batches of electrodes were tested once on the first day and tested again after every 1 to 3 weeks. Measurements were stopped when the response of the electrodes was reduced by 75% from the ideal response.

#### 2.4.3. Application for Aluminium Ion Analysis in Water Samples from the Treatment Plant 

Four types of water samples (intake, sedimentation, filtration, and clear water) were taken from a water treatment plant located at Seremban (Malaysia). The concentration of potassium ions in these water samples is low and typically within the range of 0.72 to 8.30 ppm (1.84 × 10^−5^ to 2.12 × 10^−4^ M) [40], which would not cause interference to the sensor system. This is because such concentration is low compared with the added potassium ion concentration of 0.050 M potassium ions and the concentration of potassium ions in the sample matrix remains constant. Therefore, no change in the potential of the pseudo-reference electrode. High-density polyethylene (HDPE) plastic bottles were used for water sampling. All the samples were kept in the freezer when not in use as preservation. The concentration of aluminium ions in the four treated water samples was determined using two different kinds of sensor systems, i.e., the conventional system and the all-solid-state system. For the all-solid-state system measurement, KNO_3_ was added to the water samples to ensure that the potassium ion concentration was fixed at 0.050 M KNO_3_. 

## 3. Results and Discussions

### 3.1. Performances Assessment of Potassium Ion Sensor for Pseudo-Reference Construction

High sensitivity and selectivity of the potassium ion sensor towards potassium ion are vital. It is to ensure its potential remains stable, constant, and is not influenced by the change of aluminium ion concentrations when used with an aluminium ion sensor. Therefore, an analysis of the potassium ion sensor’s sensitivity and selectivity was performed before evaluating the potassium ion sensor as a pseudo-reference electrode for the aluminium ion sensor.

The amount of selective ionophore had a significant effect on an ion sensor’s ability to determine a specific ion. An anionic site is vital for a cation-selective electrode to have a better sensitivity as it helps in rendering the perm selective of the ion-selective membrane, optimizing sensing selectivity, and reducing the bulk membrane impedance [41]. Therefore, potassium ionophore I and lipophilic anion salt, KTClPB, play a vital role in transferring potassium ion within and outside membrane pBA for good potential at the phase interface. 

**Figure 2:**
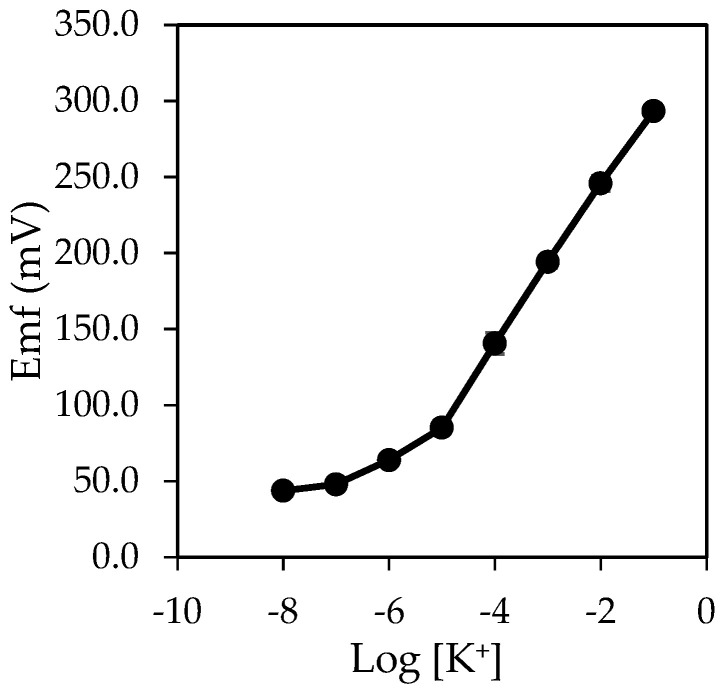
The potassium ion sensor’s potentiometric response with 1.5 mg of potassium ionophore I and 60 mol% KTClPB.

According to Figure 2, the potassium ion sensor exhibited a Nernstian response of 56.58 ± 0.06 mV/decade towards potassium ion in a linear range from 10^−5^ to 10^−1^ M. The good sensitive behavior demonstrated that the amount of potassium ionophore I and lipophilic anion salt, KTClPB (1.5 mg and 60 mol%) immobilized within the membrane pBA was optimum. Hence, the cation-exchange reactions at the membrane-solution interface were efficient, and the ion-transport process within the pBA membrane was good. Besides, the absence of plasticizer and the ability to effectively immobilized membrane components within the poly (nBA) reduces the leaching problem, and these are the reasons for a good sensor response. This response was similar to the potassium ion sensor-based pBA membrane reported by Lee et al. [32] with a sensitivity slope of 59.00 ± 0.60 mV/decade in a linear range from 10^−5^ to 10^−1^ M.

According to Table 1, it was found that most of the monovalent, divalent, and trivalent cations did not show significant interference as their average logarithm selectivity coefficient was less than −3. Selectivity depends on the complex formation between ionophore and the target ion [42,43]. The sufficient amount of ionophore within the membrane pBA enabled the maximum stable potassium ionophore I-potassium ion complex to be formed. The optimum complex distribution could also prevent the interference ions from entering the membrane, hence, gave good selectivity to the sensor [44]. Besides, the potassium ion sensor was selective towards potassium ion compared to other cations because of valinomycin as the ionophore. Valinomycin is a dodecadepsipeptide that consists of 12 amino acids and esters, which arranges alternately to form a macrocyclic molecule. Valinomycin works as a specific transporter for potassium ion by assisting the potassium ion’s movement across the membrane pBA from bulk solution into the membrane. Furthermore, the valinomycin-potassium ion complex’s stability constant is 10^6^, and this value indicates that valinomycin is suitable as a potassium ionophore for the potassium ion sensor [45]. Thus, the potassium ion sensor exhibited good selectivity towards potassium ion.

For reversibility study of potassium ion sensor, the average slope of graphs and the linear range was 56.57 ± 0.43 mV/decade in the concentration range of 10^−5^–10^−1^ M. The standard deviation (SD) and relative standard deviation (RSD) values were found to be 0.43% and 0.76%. These values showed good reversibility of the sensor. Whereas for repeatability study, a good Nernstian response, 56.89 ± 0.09 mV/decade was obtained in the concentration range of 10^−5^–10^−1^ M. The SD and RSD values were 0.09% and 0.15%, and the sensor is said to be repeatable. For the reproducibility study, the linear response obtained was in the range of 10^−5^ to 10^−1^ M, and the average slope of graphs was Nernstian, 56.58 ± 0.06 mV/decade. The SD and RSD for the average sensitivity value was 0.06% and 0.11%, which showed the sensor’s good reproducibility. Good reversibility and repeatability indicated that potassium ionophore KTClPB and I were compatible with the pBA membrane. Sufficient lipophilic of potassium ionophore I and KTClPB enabled it to be used repeatedly without leaching into aqueous samples [42,45]. Besides, for the potassium ion sensor’s design, a hydrated pHEMA with 0.1 M KNO_3_ (hydrogel) functioned as an internal electrolyte layer. With the ionic charged species’ presence within the layer, thermodynamically controlled at the interface between pBA membrane and Ag/AgCl electrodes becomes more efficient. Thus, the potassium ion sensor can display good reversibility and repeatability [46]. Simultaneously, a good reproducibility behavior showed that the amount of potassium ionophore KTClPB and I was homogenous within the pBA membrane. All the results indicate that the sensor is operational because the value of RSD for the three parameters is below the acceptable limit (<5.0%) [47].

**Figure 3:**
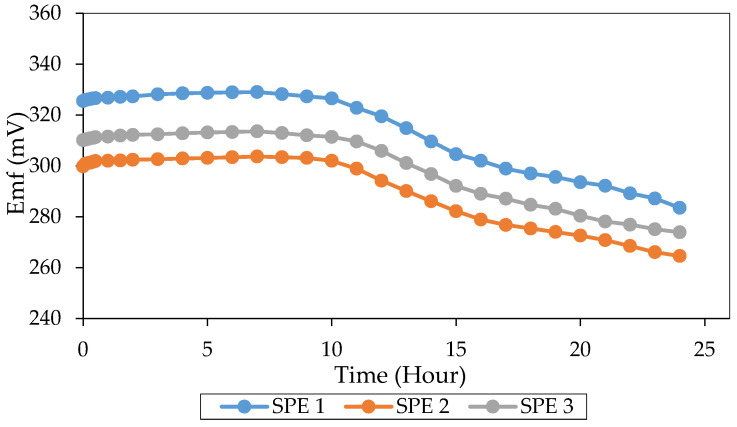
Stability study of potassium ion sensor in 0.050 M KNO_3_ solution for 24 h.

The stability of a reference electrode significantly affects the accuracy and precision of the sensor response, mainly when it performs together with an ion sensor in a continuous measurement [20]. Therefore, a potassium ion sensor with good stability is demanded because of its pseudo-reference role for aluminium ion sensors. Figure 3 shows the stability patterns of the potassium ion sensor with the change of time. For the first 10 h, the value of the potential difference revealed a small drift of 0.49 ± 0.22% with 0.20 ± 0.02 mV/h. At the 10th to the 15th hour, the potential differences exhibited a more substantial drift of 6.49 ± 0.26% with −4.14 ± 0.22 mV/h.

Meanwhile, the potential differences at the 15th to 24th hour gave a drift of 6.47 ± 0.40% (2.16 ± 0.16 mV/h). The drift rate of a suitable reference electrode should not exceed 1 mV/h [48]. Therefore, the potassium ion sensor can be said to display excellent stability in the first 10 h. A higher drift rate occurred after 10 h because of the leaching of lipophilic salt, KTClPB, into aqueous solution [29]. The lack of anion lipophilic within the membrane pBA caused the anion to enter the membrane. Thus, explained the dropped of the potential differences. However, the maximum duration of analysis using the potassium ion sensor was 30 min. Stability up to 10 h was sufficient for the overall analysis, and drift of potential differences after 10 h was acceptable.

According to Table 2, the potassium ion sensor in this work showed comparable sensitivity, selectivity, and reproducibility with the other potassium ion sensors. Therefore, a planar and solid-state potassium ion sensor with good analytical performances has been produced.

Since the potassium ion sensor would have functioned as a pseudo-reference electrode, this electrode’s stability performance was compared to the previously reported solid-state reference electrodes. By referring to Table 3, it was found that the pseudo-reference electrode in this work showed comparable duration time of stability except for the reference electrode reported by Alva et al. [29]. Compared with this electrode (5 h), this study’s pseudo-reference electrode revealed a more prolonged duration time of stability (10 h). The duration time of stability up to 10 h is sufficient for the overall analysis. It is because the maximum duration of analysis by using the pseudo-reference electrode is 30 min. However, this work’s pseudo-reference electrode demonstrated a smaller value for the drift of potential differences. Stability can be defined as the electrode’s ability to keep its potential constant during the analysis period [49]. Therefore, the pseudo-reference electrode improved its stability, and it is suitable to be studied as a solid-state reference electrode.

From the studies, the potassium ion sensor revealed good analytical performances in terms of sensitivity, selectivity, reversibility, repeatability, reproducibility, and stability. Hence, a further study assessed it as a pseudo-reference electrode for aluminium ion sensor for aluminium ion determination in an all-solid-state detection system.

### 3.2. The Performance of the All-Solid-State Aluminium Ion Analytical System 

The all-solid-state aluminium ion analytical system’s analytical performance consisted of a solid-state aluminium ion sensor, and a solid-state potassium ion sensing pseudo-reference was evaluated. 

#### 3.2.1. Effect of Concentrations of KNO_3_

**Figure 4:**
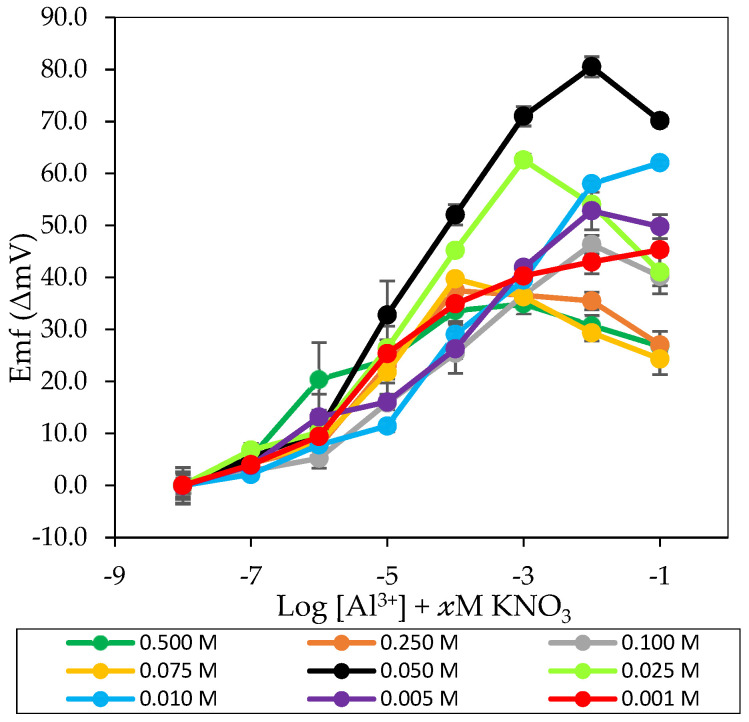
The potassium ion sensor’s potentiometric response as a pseudo-reference electrode for aluminium ion sensor in 10^−8^–10^−1^ M of aluminium ion standard solution with different concentrations of KNO_3_.

According to Figure 4 and Table 4, solution A and solution B with 0.500 M and 0.250 M KNO_3_ showed a sub-Nernstian response at a lower concentration range of Al^3+^ M. The sub-Nernstian response was due to the concentration of KNO_3_ in solution A and B had exceeded the limit for potassium ion sensor to tolerate. By referring to the linear range of potassium ion sensor (10^−5^ to 10^−1^ M), the maximum concentration of KNO_3_ was 0.1 M. Anion would enter into the membrane pBA of the potassium ion sensor with a concentration higher than 0.1 M [42]. Thus, it was found that the response was only shown at a more diluted aluminium ion concentration range, and the better response was shown in solution B with a lower KNO_3_ concentration. Whereas for solution C with 0.100 M KNO_3_, a sub-Nernstian response was also revealed, however, the linear range was displayed at a broader and more concentrated range of Al^3+^ M. Although this concentration was within the linear range of potassium ion sensor, anion interference still occurred due to the use of AlCl_3_ salts in the preparation of aluminium ion standard solution. Three moles of Cl^-^ was produced from 1 mole of AlCl_3_. Together with the anion NO_3_^-^ from KNO_3_, the presence of anions in the solution also exceeded the maximum limit of the potassium ion sensor’s anion concentration. Thus, the ion sensor became insensitive at the concentration higher than the upper limit of the linear range [39].

For solution D (0.075 M KNO_3_), a Nernstian response of 15.07 ± 0.26 mV/decade was obtained, but the linear range has fallen into the more diluted concentration range of 10^−6^–10^−4^ M Al^3+^. Anion interferences had still occurred at a higher concentration range of Al^3+^ M. However, when the KNO_3_ concentration was further lowered to 0.050 M (solution E), an excellent Nernstian response, 17.70 ± 0.13 mV/decade at a more comprehensive linear range of 10^−6^ ˗ 10^−2^ M Al^3+^ was revealed. This good sensitivity performance indicated that no occurrence of anion influx into the pBA membrane; thus, the potassium ion sensor was able to maintain its potential constant from 10^−6^ to 10^−2^ M Al^3+^ [50]. Therefore, 0.050 M KNO_3_ was suitable for the potassium ion sensor as a pseudo-reference electrode for an aluminium ion sensor.

This study was also further conducted with a lower concentration of KNO_3_ to investigate its effect on the sensor response. For solution F (0.025 M KNO_3_), a Nernstian reaction, 17.31 ± 0.26 mV/decade was obtained but at a more dilute concentration range of Al^3+^ M. This was because aluminium ions in the solution became dominant and competed with potassium ions to interfere with the potassium ion sensor. This phenomenon was supported by Alegret [51]. In a mixed solution with two kinds of ions, the ion’s presence at high concentrations will compete with the other ion for a sensor response. Besides, the sub-Nernstian response also tends to occur in such a condition. Therefore, for 0.010 to 0.001 M KNO_3_, it could be seen that a descending response with a shorter linear range was observed. The response was also shown at a more diluted Al^3+^ M concentration range. All these results were due to the dominant interference effect of aluminium ions in the low concentration of KNO_3_.

Among the nine KNO_3_ solutions, 0.050 M KNO_3_ (solution E) was the optimum concentration for the potassium ion sensor as a pseudo-reference electrode. In this concentration, the potassium ion sensor’s potential was constant and was not affected by the change in concentration of aluminium ion standard solution. Good Nernstian response and wide linear range were revealed. Therefore, this concentration was used for further study. 

#### 3.2.2. pH Effect

**Figure 5:**
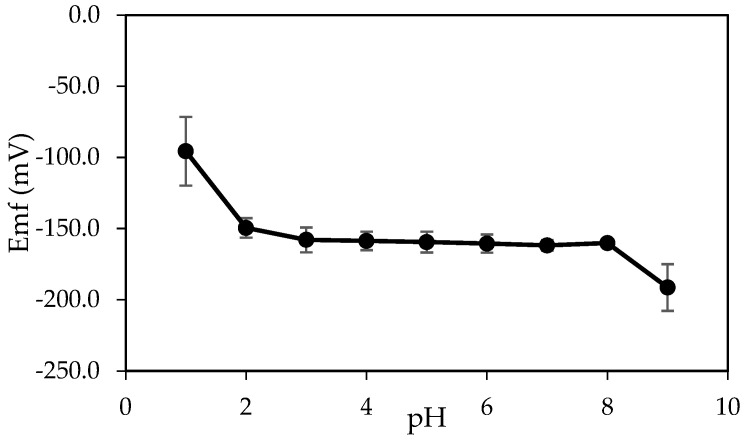
pH effect of the all-solid-state aluminium ion detection system in solutions consisted of 10^−3^ M AlCl_3_ and 0.050 M KNO_3_.

The influence of the pH of the test solution on the potential response was examined to determine the useful pH range in which the pseudo-reference electrode and aluminium ion sensor can be used. From Figure 5, the potential difference remained constant at the value of ~150 mV from pH 2.0 to 8.0. For pH lower than 2.0, the potential difference raised sharply. This difference might occur due to the response of the membrane pBA from both electrodes to H^+^ ion at a lower pH, and both ionophores lost their functional, active site as protonation of ionophores occurred [52]. Beyond the pH range, a gradual decrease in potential was also observed. This observation could be due to the formation of aluminium hydroxide species in the solution. Free aluminium ions decreased in the solution; thus, a decline of potential drift was showed [53,54]. From the results, pH 2.0 to 8.0 were considered a functional pH range for the all-solid-state miniaturized aluminium ion detection system.

#### 3.2.3. Reversibility, Repeatability, and Reproducibility

The all-solid-state aluminium ion analytical system showed a Nernstian response, 17.84 ± 0.29 mV/decade in the concentration range of 10^−6^–10^−2^ M after three reversible measurements. The SD and RSD were found to be 0.29% and 1.63%. It was noticed that this measurement system is fully reversible. Whereas for repeatability study, a good Nernstian response, 17.62 ± 0.18 mV/decade in the concentration range of 10^−6^–10^−2^ M was demonstrated. The SD and RSD values were 0.18% and 1.02%, and this system is said to be repeatable. Reproducibility was also good as all three batches of sensors demonstrated a very similar response. The linear response obtained was 17.70 ± 0.13 mV/decade in the range of 10^−6^–10^−2^ M. The SD and RSD were 0.13% and 0.73%. 

The all-solid-state aluminium ion analytical system is shown to demonstrate good reversibility, repeatability, and reproducibility. This is due to an acceptable RSD value for a working sensor system is less than 5.0% [47]. This also indicates that with the presence of 0.050 M KNO_3_ in each concentration of the standard solution of aluminium ion, the pseudo-reference electrode maintained its potential without affecting the response of the aluminium ion sensor. The good performances were due to a pseudo-reference electrode’s design with sufficient immobilization of ionophore and anion lipophilic within the pBA membrane, adequate lipophilicity of ionophores, and a balanced composition of ionophores and anion lipophilic [42,46,55].

#### 3.2.4. Stability 

**Figure 6:**
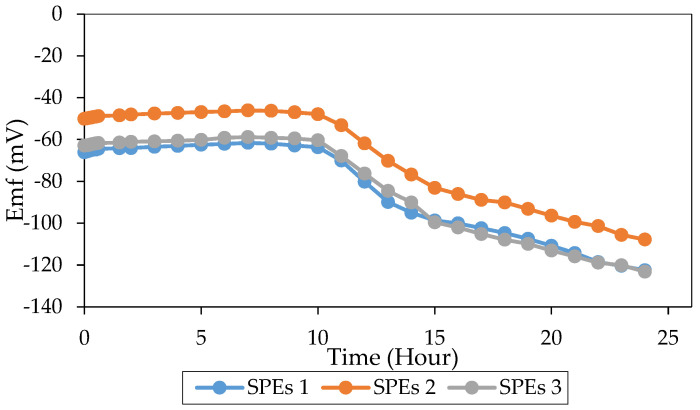
Stability study of the miniaturized system for 24 h (0.010 M AlCl_3_ and 0.050 M KNO_3_ solution).

The stability of the all-solid-state aluminium ion sensor system was also determined. Figure 6 shows the stability patterns of the system with the measurement time. For the first 10 h, the value of the system’s potential difference showed a drift of 4.00 ± 0.48% with 0.32 ± 0.01 mV/h. At the 10th to the 15th hour, the potential differences exhibited a sharp decrease of 64.44 ± 9.28% in the average of −7.48 ± 0.23 mV/h. Meanwhile, at the 15th to 24th hour, the potential differences showed a drift of 25.85 ± 3.37% (–2.74 ± 0.11 mV/h). The drift rate of a suitable electrode was reported not to exceed 1 mV/h [48]. Therefore, this system displays good stability in the first 10 h. The leaching of lipophilic salt caused a higher drift of potential differences after 10 h, KTClPB from the pseudo-reference electrode, and NaTFPB from aluminium ion sensor into aqueous solution [29]. The leaching of anion lipophilic from the membrane pBA caused the sensors to become less sensitive to their analyte ions. The anion may enter the membrane, thus, the drop of the potential differences.

#### 3.2.5. Limit of Detection and Response Time

The limit of the all-solid-state ion sensor system’s detection was found to be (2.45 ± 0.78) × 10^−7^ M (0.0066 ppm). This LOD is lower than the permissible aluminium ion level (7.41 × 10^−6^ M or 0.2000 ppm) in drinking water. Thus, it is suitable to be used as an analytical tool for aluminium ions monitoring in water samples. The time taken by the sensor to attain a steady potential is a static response time. The response time was less than 50 s for 10^−8^ to 10^−1^ M aluminium ions concentration change for this new measuring system. This response time was also similar to those reported ion sensors based on pBA membrane [9,42].

#### 3.2.6. Selectivity 

A selectivity study was performed to determine the pseudo-reference electrode’s ability to maintain its potential when used with an aluminium ion sensor in a solution containing interference ion. If the pseudo-reference electrode is not disturbed by the interference ion, a good selectivity towards aluminium ion over the other ions would be revealed. In this study, 0.010 M was selected because this concentration was within the linear range of the sensor system. According to Table 5, it was found that all interference ions did not show significant interference as their average logarithm selectivity coefficient was less than −3. Potassium ion might affect the potassium ion sensor since it was employed as a pseudo-reference electrode. However, the logarithm selectivity coefficient value for potassium ion was as low as –3.31 ± 0.23, and no interference was displayed. This observation was due to the concentration of potassium interference ion, 0.010 M when added with 0.050 M KNO_3_ from the solution, and the total concentration was changed up to 0.060 M. The disturbance produced was minimally negligible. Since all the logarithm selectivity coefficient values were less than –3, the result indicated that the pseudo-reference electrode system with an aluminium ion sensor showed an excellent selectivity towards aluminium ions. 

#### 3.2.7. Shelf Life

**Figure 7:**
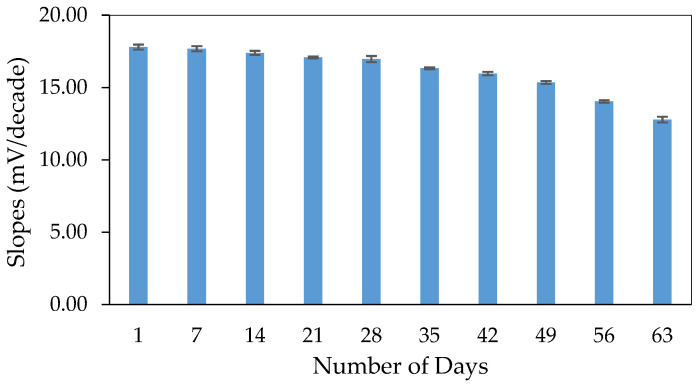
The shelf life of the all-solid-state aluminium ion sensor system.

The lifetime is another important parameter for measuring the durability of the system. During the lifetime studies (Figure 7), the aluminium sensor system’s sensitivity decreased by less than 10% after 7 weeks of storage with a linear range of 10^−6^ to 10^−2^ M Al^3+^. However, after 9 weeks, the sensitivity became sub-Nernstian of 12.79 ± 0.20 mV/decade with a narrower linear range (10^−6^ to 10^−3^ M Al^3+^). The response declined due to the active components within the pBA membrane that changed over time [55]. The leaching of the components from the membrane phase into the solution might cause this. This kind of behavior has been seen for most introduced membrane sensors [56,57]. 

#### 3.2.8. Application in Real Samples

**Figure 8:**
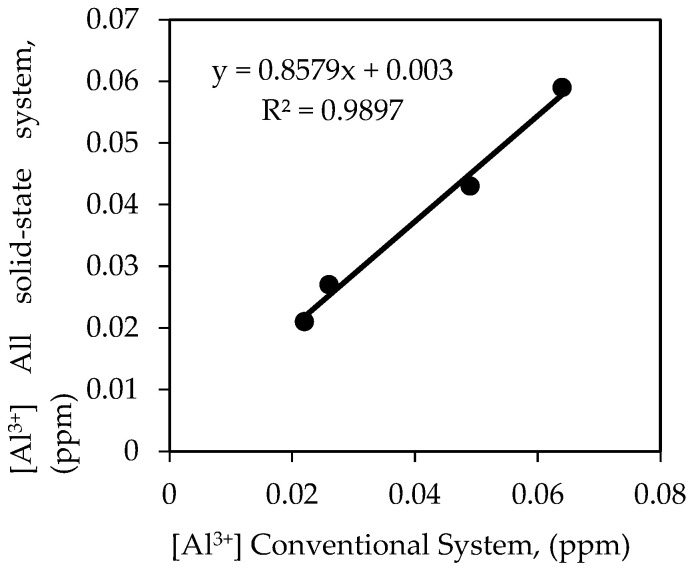
Comparison of aluminium ion concentration in four water treatment samples by using a conventional system and all-solid-state aluminium ion sensor system.

An attempt was made to analyze the concentration of an aluminium ion in real water samples. The results were also compared with the results obtained using a conventional detection system, which is the double junction standard Ag/AgCl reference electrode with the aluminium ion sensor. Table 6 shows the results of aluminium ion concentration obtained by using the all-solid-state aluminium ion sensor system and a conventional system. Based on the *t*-test statistical analysis at 95% confidence level with degrees of freedom equal to 2, t_critical_ value is 4.303, if t_calculated_ < 4.303 or t_calculated_ > −4.303, there is no significant difference between the two analytical methods [58,59,60]. From the results obtained, all the t_calculated_ values were within the range, which was—1.000, 2.449, 0.520, and 0.655. These results indicated that the aluminium ion concentration determined by the new all-solid-state aluminium ion sensor system was comparable with the value obtained by a conventional detection system. Furthermore, a good correlation relationship between the two types of systems in determining the concentration of aluminium ions in water treatment samples was also revealed in Figure 8. The correlation coefficient R was found to be 0.9948. Therefore, the new all-solid-state aluminium ion sensor system could produce comparable analytical results with the results obtained with a conventional system. These fair agreements reflect the excellent accuracy and precision of the proposed miniaturized potentiometric aluminium ion detection system.

#### 3.2.9. Comparison with Conventional Aluminium Ion Detection Systems

The new all-solid-state aluminium ion sensor system’s performances in this work were compared with the conventional system [31]. According to Table 7 and Table 8, the new system showed comparable sensitivity, linear range, response time, and selectivity with the conventional detection system. However, the new system showed a lower detection limit, improved reversibility, repeatability, reproducibility, and stability. In terms of shelf life, the new all-solid-state aluminium ion sensor system (7 weeks) was found to have a shorter life span than the conventional system (9 weeks). The shorter life span is due to the pseudo-reference electrode where the active membrane components of the solid-state potassium sensor might have leached out from the thin membrane. However, 7 weeks of shelf life was good and acceptable because membrane-based ion sensors typically have a life span of 4 to 10 weeks [61]. 

The performances of this all solid-state system also compared with the previously reported aluminium ion sensors based on different types of aluminium ionophores in PVC membrane [62,63]. According to Table 7 and Table 8, the new system showed improvement in term of detection limit over all the reported aluminium ion sensors. Besides, it also showed better selectivity over Ca^2+^, Mg^2+^, K^2+^, Na^+^ and Fe^3+^ ions. Therefore, the results indicated that the all solid-state system gives better performances over the other conventional aluminum detection systems.

## 4. Conclusions

The novelty of the aluminium ion sensor developed here is the design of an all-solid-state device. This is achieved by a solid-state aluminium ion sensor using a new ionophore and a solid-state pseudo-reference electrode, which is based on a solid-state potassium ion sensor. The all-solid-state aluminium ion sensors demonstrated analytical performance (in terms of the limit of detection, selectivity, reversibility, repeatability, reproducibility, and stability) that is comparable or better than many reported conventionally design aluminium sensors, which are not all-solid-state. In particular, the solid-state pseudo-reference electrode used with 0.050 M KNO3 could maintain a stable potential in various aluminium ion concentrations. This new system was applied for aluminium ions determination in several treated water samples, and the results are also comparable with a conventional potentiometric aluminium ion detection system. Therefore, the studies showed that using a solid-state pseudo-reference based on a potassium ion sensor had enabled a new all-solid-state aluminium ion sensor to be developed. This new system is expected to be portable and robust, particularly beneficial for on-site aluminium ion analysis, especially in water treatment plants where chemicals based on alums are used in the treatment processes extensively.

## Figures and Tables

**Table 1:** Logarithm selectivity coefficient values, $\log\;(K_{K^{+,}{\;M}^{n+}}^{\mathrm{pot}})$ of potassium ion sensor.

Types of Metal Ions (M^n+^)	$\mathrm{Log}\;(K_{K^{+,}M^{n+}}^{\mathrm{pot}})$
	Average ± SD (n = 3)
Ca^2+^	3.60 ± 0.15
Mg^2+^	−3.61 ± 0.28
Cd^2+^	−3.40 ± 0.26
Al^3+^	−3.70 ± 0.23
Ni^2+^	−3.45 ± 0.16
Pb^2+^	−3.33 ± 0.28
Cu^2+^	−3.16 ± 0.14
Zn^2+^	−3.33 ± 0.23
Hg^2+^	−3.33 ± 0.28
Na^+^	−3.04 ± 0.03
Fe^3+^	−3.06 ± 0.04

**Table 2:** Comparisons of potassium ion sensor based on potassium ionophore I in membrane pBA with the previously reported potassium ion sensors.

Type of Parameter	This Work	[32]	[11]	[44]
Slope (mV/decade)	56.58 ± 0.06	59.00 ± 0.60	56.10 ± 0.17	52.21
Linear Range (M)	10^−5^–10^−1^	10^−5^–10^−1^	10^−5^–10^−1^	10^−6^–10^−1^
Logarithm Selectivity Coefficient (SSM)	Na^+^ (−3.04 ± 0.03) Ca^2+^ (−3.60 ± 0.15) Mg^2+^ (−3.61 ± 0.28)	Na^+^ (−3.80 ± 0.05) Ca^2+^ (−4.90 ± 0.05) Mg^2+^ (–4.60 ± 0.05)	Na^+^ (−3.00 ± 0.06) Ca^2+^ (−4.50 ± 0.06) Mg^2+^ (−4.50 ± 0.09)	Na^+^ (−3.47) Ca^2+^ (−3.62) Mg^2+^ (−3.73)
Reversibility Repeatability Reproducibility (RSD%)	0.76 0.15 0.11	- - 1.00	- 0.32 0.30	- - -

**Table 3:** The comparison between the potassium ion sensor (pseudo-reference electrode) with other reported solid-state reference electrodes.

Types of Solid-State Reference Electrodes	Components of Reference Membrane	Stability (mV/hour)	Duration of Stability Study (h)	References
Potassium ion sensor pBA-pHEMA-Ag/AgCl SPE	Potassium ionophore I and KTClPB	0.20 ± 0.02	10	This work
pBA-single-walled carbon nanotubes/octadecylami-ne-SPE based on ink 7102 conductor paste	Tetradodecylammonium tetrakis-(4-chlorophenyl)- borate, KCl and AgCl	0.90 ± 0.20	10	[13]
pBA-single walled carbon nanotubes/octadecylami-ne-glassy carbon rod	Tetradodecylammonium tetrakis-(4-chlorophenyl) borate, KCl and AgCl	−1.10 ± 0.10	12	[10]
pBA-Ag/AgCl SPE	Sodium tetrakis [3,5-bis(trifloromethyl)- phenyl]borate and trioctylmethyl ammonium chloride	0.97 ± 0.04	5	[29]
Cellulose acetate-polypyrrole-carbon SPE	Sodium polyanethole sulfonate	<0.40	60	[48]
Cellulose acetate/Arabic gum-Ag/AgCl-SPE	-	0.57	72	[20]

**Table 4:** Performances of potassium ion sensor as a pseudo-reference electrode for aluminium ion sensor in aluminium ion standard solution 10^−8^–10^−1^ M with different concentrations of KNO_3_.

Type of Solutions	[KNO_3_], M	Slope (mV/Decade) ± SD, n = 3, (Al^3+^)	Linear Range (M Al^3+^)	R^2^
A	0.500	9.59 ± 0.68	10^−7^–10^−4^	0.982
B	0.250	14.88 ± 0.11	10^−6^–10^−4^	0.999
C	0.100	10.40 ± 0.36	10^−6^–10^−2^	0.999
D	0.075	15.70 ± 0.26	10^−6^–10^−4^	0.997
E	0.050	17.70 ± 0.13	10^−6^–10^−2^	0.989
F	0.025	17.31 ± 0.26	10^−6^–10^−3^	0.998
G	0.010	15.76 ± 0.64	10^−5^–10^−2^	0.996
H	0.005	12.43 ± 0.15	10^−5^–10^−2^	0.997
I	0.001	12.41 ± 0.32	10^−6^–10^−4^	0.993

**Table 5:** Logarithm selectivity coefficient values, $\log\;(K_{{\mathrm{Al}}^{3+,}{\;M}^{n+}}^{\mathrm{pot}})$ of the all-solid-state aluminium ion sensor system.

Types of Metal Ions	$\;\mathrm{Log}\;(Al_{Al^{3+,}M^{n+}}^{\mathrm{pot}})$
	Average ± SD (n = 3)
Ca^2+^	−3.42 ± 0.18
Mg^2+^	−4.04 ± 0.08
K^+^	−3.31 ± 0.23
Na^+^	−3.31 ± 0.19
Fe^3+^	−3.82 ± 0.11

**Table 6:** Quantification of ion aluminium in water treatment samples by using a conventional system with an all-solid-state system.

Types of Water Treatment Samples	The Concentration of Aluminium Ions		t-test
	Conventional System: Double Junction Conventional Ag/AgCl Reference Electrode (ppm ± SD), n = 3 [33]	All-Solid-State Aluminium Ion Sensor System with a Pseudo- Reference Electrode (Potassium Ion Sensor) (ppm ± SD), n = 3	
1	0.026 ± 0.003	0.027 ± 0.002	−1.000
2	0.049 ± 0.005	0.043 ± 0.008	2.449
3	0.064 ± 0.012	0.059 ± 0.007	0.520
4	0.022 ± 0.001	0.021 ± 0.002	0.655

t-test; t_critical_, t = 4.303 with a degree of freedom = 2; Sample code: 1—water from intake point; 2—from sedimentation tank; 3—from filtration tank; 4—final treated water.

**Table 7:** Comparison of the performances of a new all-solid-state aluminium ion sensor system with a conventional detection system and other aluminium ion sensors.

Parameters	Types of Aluminium Ion Detection System			
Types of Reference Electrodes	All-Solid-State Aluminium Ion Sensor System with a Pseudo-Reference Electrode (Potassium Ion Sensor)	Conventional Double Liquid Junction Conventional Ag/AgCl Reference Electrode [31]	Conventional Saturated Caromel Electrode Hg/Hg_2_Cl_2_/KCl [62]	Conventional Saturated Caromel Electrode Hg/Hg_2_Cl_2_/KCl [63]
Types of aluminium ionophore and matrix	1,1′-[(methylazanediyl)-bis-(ethane-2,1-diyl)]bis-[3-(naphthalen-1-yl)-thiourea] ACH, pBA membrane	1,1′-[(methylazane-diyl)-bis-(ethane-2,1-diyl)]bis[3-(naphtha-len-1-yl)thiourea] ACH, pBA membrane	2-(4,5-dihydro-1,3- imidazol-2-yl)phe-nol, PVC membrane	Glyoxal-bis-thiose-micarbazone schiff base, PVC membrane
Linear range (M Al^3+^)	1.0 × 10^−6^ –1.0 × 10^−2^	1.0 × 10^−6^–1.0 × 10^−1^	1.0 × 10^−6^–1.0 × 10^−1^	1.8 × 10^–5^–1.0 × 10^–1^
Slope (mV/decade)	17.70 ± 0.13	18.67 ± 0.56	19.30	20.10
Limit of detection (M Al^3+^)	2.45 × 10^−7^	8.07 × 10^−7^	7.00 × 10^−7^	8.70 × 10^–6^
Response time (s)	<50	35–50	10	10–15
Stability (mV/h)	0.33 ± 0.01	0.41 ± 0.02	-	-
Reversibility Repeatability Reproducibility (%)	1.63 1.02 0.73	3.77 1.22 2.99	- - -	- - -
Shelf life (Days)	49	63	120	30

**Table 8:** Comparison of the selectivity behavior of a new all-solid-state aluminium ion sensor system with a conventional detection system and other aluminium ion sensors.

	$\mathrm{Log}\;(K_{Al^{3+,}M^{n+}}^{\mathrm{pot}})\;\mathrm{Average}\;\pm \;\mathrm{SD}\;(n=3)$			
Types of Reference Electrodes	All-Solid-State Aluminium Ion Sensor System with a Pseudo-Reference Electrode (Potassium Ion Sensor)	Conventional System Double Junction Conventional Ag/AgCl Reference Electrode [31]	Conventional Saturated Caromel Electrode Hg/Hg_2_Cl_2_/KCl [62]	Conventional Saturated Caromel Electrode Hg/Hg_2_Cl_2_/KCl [63]
Types of aluminium ionophore and matrix	1,1′-[(methylazanediyl)-bis-(ethane-2,1-diyl)]-bis-[3-(naphthalen-1-yl)thiourea] ACH, pBA membrane	1,1′-[(methylazane-diyl)-bis-(ethane-2,1-diyl)]bis[3-(naphthalen-1-yl)-thiourea] ACH, pBA membrane	2-(4,5-dihydro-1,3- imidazol-2-yl)- phenol, PVC membrane	Glyoxal-bis-thio- semicarbazone Schiff base, PVC membrane
Ca^2+^	−3.42 ± 0.18	3.76 ± 0.17	−2.46	-
Mg^2+^	−4.04 ± 0.08	−4.36 ± 0.22	−2.57	−1.31
K^+^	−3.31 ± 0.19	−3.44 ± 0.15	−3.18	−2.46
Na^+^	−3.31 ± 0.23	−3.45 ± 0.08	−3.17	−2.26
Fe^3+^	−3.82 ± 0.11	−4.00 ± 0.04	−2.61	−1.45
